# Association of blood lipids with onset and prognosis of amyotrophic lateral sclerosis: results from the ALS Swabia registry

**DOI:** 10.1007/s00415-023-11630-4

**Published:** 2023-02-28

**Authors:** Sebastian Michels, Deborah Kurz, Angela Rosenbohm, Raphael S. Peter, Steffen Just, Hansjörg Bäzner, Axel Börtlein, Christian Dettmers, Hans-Jürgen Gold, Andreas Kohler, Markus Naumann, Peter Ratzka, Albert C. Ludolph, Dietrich Rothenbacher, Gabriele Nagel, Johannes Dorst, the ALS Registry Swabia Study Group

**Affiliations:** 1 Department of Neurology, Ulm University Hospital, University of Ulm, Oberer Eselsberg 45, 89081 Ulm, Germany; 2 Institute of Epidemiology and Medical Biometry, University of Ulm, Ulm, Germany; 3 Molecular Cardiology, Department of Internal Medicine II, University of Ulm, 89081 Ulm, Germany; 4 Department of Neurology, Katharinenhospital Stuttgart, Stuttgart, Germany; 5 Kliniken Schmieder Konstanz, Konstanz, Germany; 6 Klinikum am Gesundbrunnen Heilbronn, Heilbronn, Germany; 7 Department of Neurology, University Clinic Augsburg, Augsburg, Germany; 8 German Center of Neurodegenerative Diseases (DZNE), Ulm, Germany

**Keywords:** Amyotrophic lateral sclerosis, Hypermetabolism, Cholesterol, Triglycerides

## Abstract

**Background:**

To date, the role of blood lipid levels and their association with the onset and prognosis of ALS is controversial. We explored these associations in a large, population-based case–control study.

**Methods:**

Between October 2010 and June 2014, 336 ALS patients (mean age 65.7 ± 10.7; 57.7% male) and 487 sex- and age-matched controls from the same geographic region were recruited within the ALS registry in Southwest Germany. Triglycerides and cholesterol (high-density lipoprotein (HDL), low-density lipoprotein (LDL), total) were measured. The ALS cohort was followed up for vital status. Conditional logistic regression models were applied to calculate odds ratio (OR) for risk of ALS associated with serum lipid concentrations. In ALS patients only, survival models were used to appraise the prognostic value.

**Results:**

High concentration of total cholesterol (OR 1.60, 95% confidence interval (CI) 1.03–2.49, top vs. bottom quartile), but not HDL, LDL, LDL–HDL ratio, or triglycerides, was positively associated with the risk of ALS. During the median follow-up time of 88.9 months, 291 deaths occurred among 336 ALS patients. In the adjusted survival analysis, higher HDL (HR 1.72, 95% CI 1.19–2.50) and LDL cholesterol levels (HR 1.58, 95% CI 1.11–2.26) were associated with higher mortality in ALS patients. In contrast, higher triglyceride levels were associated with lower mortality (HR 0.68, 95% CI 0.48–0.96).

**Conclusion:**

The results highlight the importance to distinguish cholesterol from triglycerides when considering the prognostic role of lipid metabolism in ALS. It further strengthens the rationale for a triglyceride-rich diet, while the negative impact of cholesterol must be further explored.

**Supplementary Information:**

The online version contains supplementary material available at 10.1007/s00415-023-11630-4.

## Introduction

Amyotrophic lateral sclerosis (ALS) is the most frequent motor neuron disease and is characterized by progressive degeneration of both upper and lower motor neurons. Patients suffer from hypercatabolism [1], which occurs years before the onset of motor symptoms [2, 3]. Population-based studies reported that weight loss was present in 67.5% of all ALS patients at the time of diagnosis [4]. Various studies have shown that catabolism is a strong negative prognostic factor [5–7] and that high-caloric nutrition favorably influences the course of the disease [8–11]. ALS patients exhibit an increased energy expenditure at rest [1, 10, 12, 13], which might be related to hypothalamic [14, 15] and/or mitochondrial dysfunction [16, 17].

Interestingly, patients with ALS frequently exhibit increased serum lipid and cholesterol levels [18–21]. To date, the cause of these changes remains enigmatic, since ALS patients tend to be lean at diagnosis [20] and exercise more [22, 23]. Therefore, the influence and prognostic significance of lipid metabolism in ALS is controversial. High plasma levels of cholesterol have been suggested to be neuroprotective and are associated with increased survival by some studies [18–20, 22]. However, other data suggest that accumulation of cholesterol and its metabolites mediate oxidative stress in motor neurons [24, 25] and may increase the risk of developing ALS [26]. Hypertriglyceridemia has more consistently been associated with prolonged survival [18, 19]. However, previous studies show a large heterogeneity regarding the association between lipid profiles and clinical phenotypes [27]. We suspect that this is partially driven by methodological aspects, as most of the existing literature relies on retrospective, hospital-based data, which are prone to selection and recall bias as well as confounding.

Knowledge about the effect of lipid metabolism on risk and prognosis of ALS is important because it implies direct clinical consequences, such as dietary recommendations, composition of prescribed high-caloric food supplements, and the identification of potential future therapeutic targets. Therefore, our aim was to examine the associations of total cholesterol, high-density lipoprotein (HDL), low-density lipoprotein (LDL), and triglycerides with risk of ALS and mortality within the ALS cohort in a large, well-characterized, population-based study sample.

## Methods

### Study population and design

The ALS registry Swabia is a population-based clinical-epidemiological registry in a defined geographic region in the South-West of Germany (details see [28]). The catchment area has approximately 8.4 million inhabitants. The aim of the registry is to collect data on all newly diagnosed ALS patients in Swabia. Consequently, the registry provides estimates of epidemiological variables, such as incidence, and describes the natural history of ALS, including survival status. It further allows for the investigation of risk factors for ALS by means of a registry-based case–control study.

Beginning October 01, 2010, all newly diagnosed ALS patients were registered prospectively. ALS patients were defined by the diagnosis of possible, probable or definite ALS according to the revised El Escorial criteria [29].

Additionally, all newly diagnosed and registered ALS patients between October 01, 2010 and December 31, 2014 were asked to provide written informed consent to participate in a population-based case–control study investigating risk factors for ALS. For each case, two sex- and age-matched healthy control subjects from the same geographic region were randomly selected. For this purpose, a random sample of potential control subjects was acquired from the general population as registered in the regional registry office of the respective geographic region. Potential healthy control subjects were invited by mail to participate. After written informed consent, study nurses visited patients and healthy controls for an identical standardized interview and blood sampling. Response rate was 65% in patients and 19% in controls.

ALS patients were actively followed up annually through a standardized interview. For survival status, the registration offices were contacted annually, and if a patient died, the date of death was received (last systematic mortality update in December 2020).

### Biomarker measurement

HDL, LDL, total cholesterol, and triglycerides (mmol/l) were determined from serum samples. Blood was drawn at least 1 h after the last food intake in 90.8% of the ALS cohort and 86.2% of the healthy control group (details see Table 1). Laboratory analyses were performed in the year 2019 in a blinded fashion at the central laboratory of the Department of Clinical Chemistry, Ulm University Medical Clinic, according to accredited and standardized routine methods (triglycerides photometrically on the Cobas c system; HDL, LDL, and total cholesterol enzymatically on the Cobas c system; both from Roche Diagnostics, Basel, Switzerland).

**Table 1:** Characteristics of ALS patients and matched controls

	*N*_Cases_	ALS-cases	*N*_Controls_	Control subjects
Case–control study	336		487	
Age (years), mean (SD)	336	65.7 (10.7)	487	66.1 (10.0)
Sex	336		487	
Male, *N* (%)		194 (57.7)		294 (60.4)
School education, *N* (%)	336		485	
< 10th grade		187 (54.7)		215 (44.3)
≥ 10th grade		149 (44.3)		270 (55.7)
Smoking	331		485	
Ever, *N* (%)		159 (48.0)		238 (49.1)
BMI (kg m^−2^), mean (SD)	336	24.5 (4.0)	485	26.5 (4.1)
Overweight (≥ 25 kg m^−2^), *N* (%)		136 (40.5)		289 (59.6)
Family history of ALS, *N* (%)	330		487	
Positive		15 (4.6)		3 (0.6)
Occupational work intensity, *N* (%)	327		483	
Light (mainly sitting)		115 (35.1)		248 (51.3)
Moderate (standing and walking)		141 (43.1)		173 (35.8)
Heavy (physically demanding)		71 (21.8)		62 (12.9)
Time between last meal and blood sampling (h), *N* (%)	336		487	
≤ 1		31 (9.2)		67 (13.8)
> 1–5		264 (78.6)		367 (75.4)
> 5–10		22 (6.5)		20 (4.1)
> 10		15 (4.5)		21 (4.3)
Missing		4 (1.5)		12 (2.5)
HDL (mmol L^−1^), median (IQR)	336	1.5 (1.2–1.8)	487	1.5 (1.2–1.8)
LDL (mmol L^−1^), median (IQR)	335	3.2 (2.7–3.9)	487	3.3 (2.7–3.9)
LDL–HDL ratio, median (IQR)	335	2.1 (1.7, 2.8)	486	2.4 (1.8, 2.8)
Total cholesterol (mmol L^−1^), median (IQR)	336	5.8 (5.0–6.6)	486	5.6 (4.9–6.4)
Triglycerides (mmol L^−1^), median (IQR)	336	1.7 (1.2–2.5)	487	1.7 (1.2–2.5)
Lipid-lowering medication, *N* (%)	336	38 (11.3)	487	97 (19.9)
Statins	336	36 (10.7)	487	96 (19.7)
Others (fibrates, etc.)	336	2 (0.6)	487	2 (0.4)
Antidiabetic medication, *N* (%)	336	26 (7.7)	487	37 (7.6)
Metformin	336	20 (6.0)	487	34 (7.0)
Other oral antidiabetics	336	4 (1.2)	487	14 (2.9)
Insulin	336	6 (1.8)	487	4 (0.8)

Demographic data, lipid profiles and co-medication for ALS patients and matched healthy controls

*BMI* body mass index, *HDL* high-density lipoprotein, *LDL* low-density lipoprotein, *ALSFRS-R* ALS functional rating scale revised

### Covariates

Data on body mass index (BMI), smoking status, comorbidities, lifestyle, school education, and use of lipid-lowering drugs (statins, fibrates) were collected at baseline assessment in ALS patients and healthy controls during a standardized, questionnaire-based interview. ALS-specific data including ALS functional rating scale revised (ALS-FRS-R), disease duration, site of onset (spinal/bulbar), and family history of ALS were recorded. We calculated the pre-baseline decline of ALS-FRS-R points per month as marker of disease progression based on the formula: (48-ALS-FRS-R Score at the first visit)/(months between disease onset and first visit). The disease onset was defined as the patient-reported time of first symptoms, i.e., first paresis in spinal-onset ALS or speech/swallowing disturbances in bulbar-onset ALS.

### Ethics statement

International, national, and state rules were applied for the implementation of the ALS registry Swabia. We obtained ethical approval of the ethical committees of Ulm University (reference # 11/10) and the regional medical associations (Landesärztekammer Baden-Württemberg reference # B-F-2010-062 and Landesärztekammer Bayern reference # 7/11300).

### Statistical analysis

Sociodemographic data, lifestyle, laboratory results, comorbidities, and clinical characteristics were analyzed descriptively. Generalized linear models were used to assess the association between sociodemographic/clinical variables and serum levels of HDL, LDL, LDL-HDL ratio, total cholesterol, and triglycerides. Models were controlled for case–control status, sex, and age.

Conditional logistic regression was used to calculate crude and multivariable adjusted odds ratios (ORs) and 95% confidence intervals (95%-CIs) for the association between serum quartiles of HDL, LDL, LDL-HDL ratio, total cholesterol, and triglycerides with onset of ALS. Quartile cut points were calculated based on the distribution in controls (Suppl. Table 1).

In the case–control study, logistic regression models were conditioned on sex and age-groups and additionally adjusted for relevant covariates (potential confounders) including BMI, smoking (ever), self-reported diabetes mellitus, occupational work intensity, educational attainment, and family history of ALS. The form of the associations was assessed using restricted cubic splines with knots at the 5, 35, 65, and 95% percentiles. We log-transformed the variable triglyceride as it was skewed slightly to the right. For graphical representation of the results, the variable was back transformed. The models are based on data with a full set of covariates.

In the ALS cohort, we investigated the prognostic value of HDL, LDL, LDL-HDL ratio, total cholesterol, and triglycerides on overall survival as defined by the time between onset of first paresis and death/tracheostomy (last systematic mortality update in December 2020). We performed Cox proportional hazard regression models to calculate hazard ratios (HRs) and 95% CIs by quartiles of HDL, LDL, LDL-HDL ratio, total cholesterol, and triglycerides. Model entry for observed survival time was time of first visit (baseline visit) to adjust for immortal time bias [30, 31]. Cox models were adjusted for sex, age, diagnostic delay, site of onset, and ALS-FRS-R at baseline as the main independent prognostic factors. In a second step, we additionally adjusted for BMI, and smoking (ever). We checked for effect modification between all exposure variables and sex in all fully adjusted models, respectively. If effect modification was detected, the analysis was stratified by sex. We also calculated a Pearson correlation coefficient for self-reported weight loss and HDL concentration in serum.

Sensitivity analyses were performed by excluding all ALS subjects with revised El Escorial criteria ≤ 2 (“clinically possible”) and excluding participants taking lipid-lowering or antidiabetic medications. All provided p-values are two-sided. The significance level was set as *p* < 0.05. Unless otherwise indicated, the OR and HR reported refer to the top quartile compared to the lowest quartile. The analyses were performed using SAS^®^ 9.4 (The SAS Institute, Cary, NC, USA).

## Results

Overall, 336 patients with ALS and 487 matched controls were included in the case–control study (Table 1). The mean age was 65.7 ± 10.7 years in the ALS and 66.1 ± 10.0 years in the control group. The ALS cohort had a lower mean BMI of 24.5 ± 4.0 kg*m^−2^ compared to controls (26.5 ± 4.1 kg*m^−2^, *p* < 0.001). Fifteen patients (4.6%) had a positive family history of ALS compared to three control subjects (0.6%). The ALS cohort had lower levels of school education (44.3 vs. 55.7%) and a higher proportion of physically demanding work (21.8 vs. 12.9%) related to their occupation.

Antidiabetic medication was equally frequent in patients and controls (7.7 vs. 7.6%). We noted a lower prevalence of lipid-lowering medication intake of 11.3% in the ALS cohort compared to 19.9% in controls (*p* = 0.001). Serum concentrations of HDL, LDL, LDL-HDL ratio, total cholesterol, and triglycerides were similarly distributed among patients and controls.

Among the ALS cohort, the median pre-baseline disease progression was 0.9 (0.5–1.5) ALS-FRS-R points lost per month.

### Case–control study

**Fig. 1:**
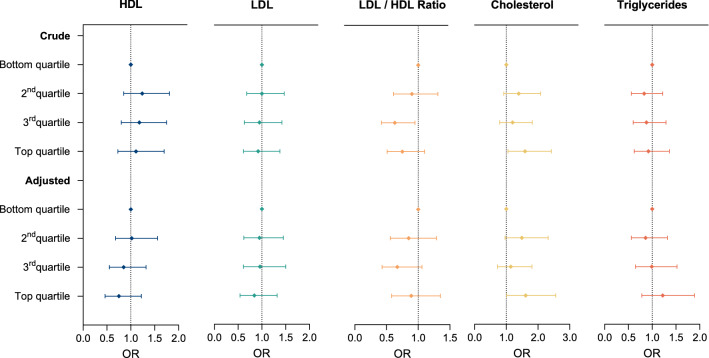
Forest plot showing associations of cholesterol and triglycerides with risk of ALS (case–control study). Lipid metabolism and risk for ALS based on values of cases and controls. Crude = conditioned on sex and age group. Adjusted = additionally adjusted for educational attainment, occupational work intensity, smoking (ever), family history of ALS, body mass index (BMI), and self-reported diabetes mellitus. *HDL* high-density lipoprotein; *LDL* low-density lipoprotein, *OR* odds ratio

In the case–control study, a higher risk for ALS was observed for the top quartile of total cholesterol (OR 1.60; 95% CI 1.03–2.49) (Fig. 1, Suppl. Table 2). No association with ALS risk was found for LDL, HDL, LDL-HDL ratio, or triglycerides. Sex was a significant modifier of cholesterol associated risk for ALS (p = 0.035). The risk for developing ALS with increased cholesterol levels was more pronounced in male subjects with an OR of 2.76 (1.48–5.14) in the top quartile (Suppl. Table 3).

### Survival analysis

In the ALS case cohort, during the median follow-up of 88.9 months, 291 deaths occurred among 336 ALS participants (Table 2). The survivor group was characterized by more men (77% vs. 54.6%), a higher mean BMI (25.4 ± 4.3 vs. 24.3 ± 4.0 kg/m^2^), and a higher median ALS-FRS-R (43.0 vs. 38.0).

**Table 2:** Patient characteristics by survival status

Clinical characteristics in the cohort of ALS cases		
Site of onset, *N* (%)		
Bulbar	336	109 (32.4)
Spinal		227 (67.6)
Revised El Escorial criteria, *N* (%)	336	
Clinically suspected		65 (19.4)
Clinically possible		34 (10.1)
Clinically probable		92 (27.4)
Laboratorysupported, probable		117 (34.8)
Clinically definite		28 (8.3)
ALS-FRS-R, median (Q1, Q3)	335	39.0 (34.0, 42.0)
Diagnostic delay (month), median (Q1, Q3)	336	5.0 (2.6, 9.1)
Invasive ventilation, *N* (%)	336	13 (3.9)
Pre-baseline ALS-FRS-R progression rate (points lost per month), median (IQR)	335	0.9 (0.5–1.5)

Characteristics of ALS cases (*N* = 335) with mortality follow-up by survival status

*BMI* body mass index, *ALS-FRS-R ALS* functional rating scale revised, *HDL* high-density lipoprotein, *LDL* low-density lipoprotein

**Fig. 2:**
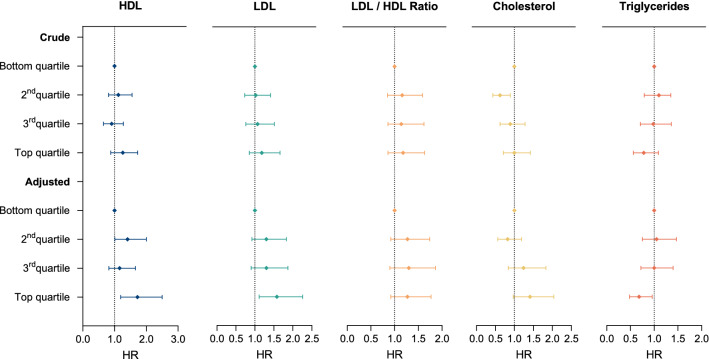
Forest plot showing associations of cholesterol and triglycerides with risk of death in ALS patients. Effect of lipid metabolism on survival in the ALS cohort. Crude = adjusted for sex and age groups. Adjusted = additionally adjusted for site of onset, and ALS functional rating scale revised (ALS-FRS-R). *HDL* high-density lipoprotein, *LDL* low-density lipoprotein, *HR* hazard ratio

Increased HDL (HR 1.72, 95% CI 1.19–2.50), and LDL (HR 1.58, 95% CI 1.11–2.26) were associated with increased mortality (Fig. 2, Suppl. Table 4). Higher triglyceride levels (top quartile), on the contrary, were associated with longer survival (HR 0.68, 95% CI 0.48–0.96). After additional adjustment for BMI and smoking, the detrimental effects of HDL and LDL were still significant (HDL: HR 1.54, 95% CI 1.05–2.27, LDL: HR 1.57, 95% CI 1.10–2.26), while the beneficial effect of triglycerides was not (HR 0.72, 95% CI 0.50–1.30, Suppl. Table 4), indicating that the life-prolonging effect of high triglycerides was partly explained by the associated higher BMI. HDL levels did not correlate with patient-reported weight loss pre-baseline during the preceding 3 months (*r* = 0.033, *p* = 0.573).

## Discussion

In this population-based study including 336 ALS patients and 487 matched controls, we found evidence for an association between elevated total cholesterol serum levels and ALS risk, but not for HDL, LDL, or triglycerides. Furthermore, high triglycerides may be associated with longer survival, whereas higher levels of cholesterol were associated with a higher mortality.

Despite the   frequently observed weight loss, hyperlipidemia is a common phenomenon in ALS patients. Cholesterol, lipoproteins (HDL and LDL), and triglycerides are crucial components of lipid metabolism and are essential for a variety of cell functions. Cholesterol, an unsaturated alcohol of the steroid family, is mainly synthesized and recycled in the liver. Its primary function is to maintain the integrity and to modulate the fluidity of cell membranes, as well as serving as a precursor for the synthesis of vitamin D and all steroid hormones. It also plays a role in cell signaling processes affecting various ion channels [32] and modulates excitatory synaptic transmission [33].

Our analysis revealed a strong association of increased cholesterol levels with both risk and mortality in ALS. In the literature, a plethora of examples link signaling pathways involved in ALS pathology to lipid metabolism [25]. A pathological hallmark of ALS is the presence of cytoplasmic inclusion bodies consisting of phosphorylated transactive response DNA-binding protein 43 (pTDP-43). Of note, TDP-43 was found to directly modulate expression levels of SREBF2, the master transcription factor of cholesterol biosynthesis [34]. Besides SREBF2, cholesterol metabolism is controlled by the liver x receptor (LXR), which was found to be a modifier for age of onset and survival in ALS patients [35].

Another possible explanation for alterations of the metabolic profile might be the involvement of the hypothalamic circuit in ALS [36]. An MRI-based study showed that ALS patients developed atrophy of the anterior and posterior hypothalamus compared to a control cohort [14]. Interestingly, these changes were also observed in presymptomatic carriers of pathogenic ALS mutations [14]. Histopathologically, there is parallel evidence of changes in the hypothalamus and hypophysis: toxic protein aggregations of pTDP-43 and dipeptide repeats in C9orf72-related ALS and ALS with frontotemporal dementia (ALS-FTD) have been found in the hypothalamic–pituitary axis [37, 38]. These structural changes could contribute to disturbances in hypothalamic hormone levels and, consequently, to alterations of lipid metabolism. Indeed, patients with ALS frequently exhibit reduced levels of growth hormone (GH) [39, 40] and alterations of the melanocortin pathway [15]. Under physiological conditions, GH stimulates the hepatic LDL receptor, leading to reduced total cholesterol and LDL levels [41]. Thus, it may be hypothesized that hypercholesterolemia might reflect incipient changes related to the underlying ALS pathology. Recent findings from large genome-wide association studies (GWAS) further support these results by providing evidence that elevated LDL [42] and total cholesterol levels [43] were risk factors for ALS.

With regard to cholesterol-associated disease risk, sex emerged as an important effect modifier, resulting in a significantly increased risk for ALS at high cholesterol levels in male subjects. Epidemiological and experimental studies have shown gender differences with regard to susceptibility to ALS and disease progression, suggesting a lower risk of developing ALS in women [44, 45]. Several studies attribute a protective effect to the female sex hormones estrogen and progesterone [46]. Interestingly, both are known to modulate lipid metabolism and cholesterol composition, which may explain the sex differences in our study.

In our survival analysis, increased levels of HDL, LDL and total cholesterol showed a positive association with overall mortality in ALS patients. These results were robust even after adjustment for established prognostic factors and clinical characteristics. Thus, we could not reproduce the results of earlier observational studies that had associated hypercholesterolemia with prolonged survival time [18–20, 22]. A possible explanation could be a lack of adjustment for cofactors in previous studies [47]. In particular, a high BMI is an independent positive prognostic factor which is frequently associated with hypercholesterolemia [3, 48] and therefore could falsely suggest a beneficial effect of hypercholesterolemia on overall survival. Of note, other studies found no association between cholesterol levels and mortality [48, 49]. Recently, a meta-analysis and population-based study by Van Mantgem et al. also showed that higher HDL levels were associated with reduced survival. However, the authors also demonstrated that elevated HDL concentrations negatively correlated with BMI, and HDL may therefore represent an epiphenomenon of weight loss [50]. In our study, we could not find a correlation between HDL and BMI.

In contrast to cholesterol, we observed a significantly increased survival for patients with higher triglycerides. In the general population, however, increased triglycerides levels are associated with increased mortality due to an increased incidence of cardiovascular events [51]. Consistent with our results, previous retrospective studies had shown a positive association between higher triglyceride levels and prolonged survival [18, 19]. This effect was no longer significant after adjusting for BMI and smoking, indicating that this effect may be partly explained by the associated higher BMI as a known independent positive prognostic factor.

Triglycerides are produced from lipogenesis in the endoplasmic reticulum of cells (mainly hepatocytes and adipocytes) by esterification of fatty acids to glycerol and are also largely taken up with food. The primary function of triglycerides is to store and transport energy in the form of fatty acids within cells and to provide energy sources in a state of starvation and glucose shortage via conversion to either glucose or ketone bodies [52]. Thus, high triglyceride levels could potentially counteract ALS-related hypermetabolism and increased energy expenditure [1, 10, 12, 13, 53]. Consistently, beneficial effects have been reported for interventions with high-fat nutritional supplements, including prolonged survival in fast progressing patients, decreased decline of motor function, stabilization of body weight, and reduction of serum neurofilament light chain levels [8, 9, 54]. Even after adjustment for BMI as a potential confounding factor, our data still suggests a significant trend towards reduced mortality associated with higher levels of triglycerides. Thus, in summary, the results support the concept of an anti-catabolic, high-caloric, and fat-rich therapy.

### Strength and limitations

The strengths of the study are: the study was based on a large prospective population-based cohort that was matched for sex, age, and area of residency. In addition, we were able to obtain prospective long-term data and assessed potential confounders by stepwise adjusted analysis. As a limiting factor, the genetic background for causative ALS mutations were not obtained from the ALS cohort.

## Conclusion

In summary, our data suggest a complex association of lipid metabolism with risk and prognosis of ALS. Based on our results, it is essential to carefully distinguish between cholesterol and triglycerides, taking into account their different physiological roles.

On the one hand, the results suggest a negative effect of increased cholesterol levels on survival as well as an association between higher cholesterol levels and an increased risk of ALS. These associations might be based on the interaction between TDP-43 and cholesterol biosynthesis, hypothalamic alterations, and/or a genetic overlap between cholesterol metabolism and ALS.

On the other hand, we found further evidence for a beneficial effect of increased triglyceride levels on survival, strengthening the rationale for an anti-catabolic therapeutic approach and highlighting the importance of triglycerides as a source of energy to counter hypermetabolism and weight loss in ALS.

## Supplementary Information

Below is the link to the electronic supplementary material.

Supplementary file1 (DOCX 28 KB)

Supplementary file2 (DOCX 19 KB)

## Data Availability

Individual participant data that underlie the results reported in this article, after de-identification (text, tables, and figures) will be available. Data will be available beginning 3 months and ending 5 years following article publication. Data will be shared with researchers who provide a methodologically sound proposal. Data will be shared for analyses to achieve the aims in the approved proposal. Proposals should be directed to johannes.dorst@uni-ulm.de; to gain access, data requestors will need to sign a data access agreement.
