# Supplementary material for: Association of blood lipids with onset and prognosis of amyotrophic lateral sclerosis: results from the ALS Swabia registry
Source: J Neurol. 2023 Feb 28;270(6):3082–90. doi: 10.1007/s00415-023-11630-4 (PMC10193299; doi:10.1007/s00415-023-11630-4)
Supplement: Supplementary file 1 — Supplementary file1 (DOCX 28 KB) [file 415_2023_11630_MOESM1_ESM.docx]

**Supplemental Tables and Figures**

**Supplemental Table 1** Cut-offs for biomarker quartiles

| N=487 controls | **HDL**  **(mmol L^-1^)** | **LDL**  **(mmol L^-1^)** | **Triglyceride (mmol L^-1^)** | **Cholesterol (mmol L^-1^)** | **LDL/HDL-Ratio** |
| --- | --- | --- | --- | --- | --- |
| Bottom quartile | -1.20 | -2.70 | -1.20 | -4.90 | -1.80 |
| 2^nd^ quartile | >1.20-1.50 | >2.70-3.30 | >1.20-1.70 | >4.90-5.60 | >1.80-2.30 |
| 3^rd^ quartile | >1.50-1.80 | >3.30-3.90 | >1.70-2.50 | >5.60-6.40 | >2.30-2.80 |
| Top quartile | >1.80 - | >3.90 | >2.50- | >6.40- | >2.80- |

Table shows the cut-offs for the quartiles of each parameter used in the analysis. The cut-offs are based on the values of controls.

**Supplement Table 2.** Associations of cholesterol and triglycerides with risk of ALS (case control study).

|  |  | **HDL**  Odds ratio  (95%-CI) | **LDL**  Odds ratio (95%-CI) | **Cholesterol**  Odds ratio  (95%-CI) | **Triglycerides**  Odds ratio (95%-CI) | **LDL/HDL Ratio** Odds ratio  (95%-CI) |
| --- | --- | --- | --- | --- | --- | --- |
| Crude (N_Cases_=336, N_Controls_=487^)^ | |  |  |  |  |  |
|  | Bottom quartile | (ref.) 1.00 | (ref.) 1.00 | (ref.) 1.00 | (ref.) 1.00 | (ref.) 1.00 |
|  | 2^nd^ quartile | 1.24 (0.85, 1.81) | 1.00 (0.68, 1.47) | 1.39 (0.92, 2.08) | 0.83 (0.56, 1.22) | 0.90 (0.61, 1.31) |
|  | 3^rd^ quartile | 1.18 (0.80, 1.75) | 0.95 (0.63, 1.42) | 1.20 (0.79, 1.82) | 0.88 (0.60, 1.29) | **0.63 (0.42, 0.95)** |
|  | Top quartile | 1.11 (0.73, 1.70) | 0.92 (0.61, 1.38) | **1.59 (1.05, 2.42)** | 0.92 (0.62, 1.36) | 0.75 (0.51, 1.10) |
| Per 0.5 mmol/l increase | | 1.06 (0.90, 1.25) | 0.99 (0.92, 1.07) | 1.05 (0.98, 1.12) | 0.98 (0.75, 1.30)* | 0.95 (0.87, 1.03) |
|  | |  |  |  |  |  |
| Adjusted (N_Cases_=309, N_Controls_=468) | |  |  |  |  |  |
|  | Bottom quartile | (ref.) 1.00 | (ref.) 1.00 | (ref.) 1.00 | (ref.) 1.00 | (ref.) 1.00 |
|  | 2^nd^ quartile | 1.02 (0.68, 1.56) | 0.95 (0.62, 1.45) | 1.49 (0.96, 2.32) | 0.86 (0.56, 1.32) | 0.85 (0.56, 1.29) |
|  | 3^rd^ quartile | 0.85 (0.55, 1.32) | 0.96 (0.61, 1.50) | 1.14 (0.72, 1.81) | 0.99 (0.65, 1.52) | 0.67 (0.43, 1.06) |
|  | Top quartile | 0.75 (0.46, 1.22) | 0.84 (0.54, 1.32) | **1.61 (1.01, 2.56)** | 1.22 (0.78, 1.89) | 0.89 (0.58, 1.35) |
| Per 0.5 mmol/l increase | | 0.90 (0.74, 1.09) | 0.97 (0.89, 1.06) | 1.03 (0.96, 1.11) | 1.19 (0.88, 1.62)* | 0.99 (0.90, 1.09) |
|  | |  |  |  |  |  |

*per 1% increase in serum triglyceride

Lipid metabolism and risk for ALS based on values of cases and controls.

crude = conditioned on sex and age groups

adjusted = additionally adjusted for educational attainment, occupational work intensity, smoking (ever), family history of ALS, body mass index (BMI), and self-reported diabetes mellitus

HDL = high density lipoprotein; LDL = low density lipoprotein

**Supplement Table 3:** Lipid metabolism (cholesterol) and risk of ALS stratified by sex (case control study).

|  |  | **Cholesterol in**  **males**  Odds ratio  (95%-CI) | **Cholesterol in females**  Odds ratio  (95%-CI) |
| --- | --- | --- | --- |
| **Crude**  Male: N_Cases_=194, N_Controls_=294  Female: N_Cases_=142, N_Controls_=193 | |  |  |
|  | Bottom quartile | (ref.) 1.00 | (ref.) 1.00 |
|  | 2^nd^ quartile | 1.41 (0.87, 2.30) | 1.33 (0.63, 2.80) |
|  | 3^rd^ quartile | 0.93 (0.55, 1.58) | 1.57 0.76, 3.23) |
|  | Top quartile | **2.25 (1.29, 3.90)** | 1.22 (0.61, 2.44**)** |
| Per 0.5 mmol/l increase | | 1.09 (1.0, 1.19) | 1.0 (0.90, 1.10) |
| **Adjusted**  Male: N_Cases_=177, N_Controls_=287  Female: N_Cases_ =132, N_Controls_=181 | |  |  |
|  | Bottom quartile | (ref.) 1.00 | (ref.) 1.00 |
|  | 2^nd^ quartile | 1.47 (0.86, 2.54) | 1.44 (0.64, 3.25) |
|  | 3^rd^ quartile | 0.97 (0.54, 1.75) | 1.24 (0.56, 2.75) |
|  | Top quartile | **2.76 (1.48, 5.14**) | 1.03 (0.48, 2.21) |
| Per 0.5 mmol/l increase | | **1.11 (1.01, 1.23)** | 0.95 (0.86, 1.06) |

Lipid metabolism and risk for ALS based on values of cases and controls.

Crude = conditioned age group

Adjusted = additionally adjusted for educational attainment, occupational work intensity, smoking (ever), family history of ALS, body mass index (BMI), and self-reported diabetes mellitus

**Supplement Table 4**: Associations of cholesterol and triglycerides with risk of death in ALS patients

|  | **HDL**  Hazard ratio (95%-CI) | **LDL**  Hazard ratio (95%-CI) | **Cholesterol**  Hazard ratio (95%-CI) | **Triglycerides**  Hazard ratio (95%-CI) | **LDL/HDL Ratio** Hazard ratio (95%-CI) |
| --- | --- | --- | --- | --- | --- |
| Crude  (N_Deceased_=287, N_Survived_=43 |  |  |  |  |  |
| Bottom quartile | (ref.) 1.00 | (ref.) 1.00 | (ref.) 1.00 | (ref.) 1.00 | (ref.) 1.00 |
| 2^nd^ quartile | 1.12 (0.81, 1.55) | 1.02 (0.73, 1.41) | **0.62 (0.43, 0.89)** | 1.10 (0.79, 1.35) | 1.16 (0.85, 1.59) |
| 3^rd^ quartile | 0.91 (0.65, 1.28) | 1.07 (0.76, 1.51) | 0.89 (0.62, 1.28) | 0.98 (0.71, 1.36) | 1.14 (0.80, 1.62) |
| Top quartile | 1.26 (0.88, 1.73) | 1.18 (0.85, 1.66) | 1.00 (0.71, 1.42) | 0.78 (0.56, 1.09) | 1.18 (0.86, 1.63) |
| Per 0.5 mmol/l increase | 1.03 (0.89, 1.19) | 1.03 (0.97, 1.10) | 1.02 (0.97, 1.08) | 0.97 (0.92, 1.02) | 1.02 (0.95, 1.10) |
|  |  |  |  |  |  |
|  |  |  |  |  |  |
| *Adjusted (N*_Deceased_*=282, N_Survived_=42)* |  |  |  |  |  |
| Bottom quartile | (ref.) 1.00 | (ref.) 1.00 | (ref.) 1.00 | (ref.) 1.00 | (ref.) 1.00 |
| 2^nd^ quartile | 1.38 (0.98, 1.94) | 1.26 (0.88, 1.79) | 0.79 (0.54, 1.18) | 1.07 (0.76, 1.51) | 1.21 (0.88, 1.67) |
| 3^rd^ quartile | 1.10 (0.77, 1.58) | 1.26 (0.87, 1.82) | 1.19 (0.81, 1.77) | 1.04 (0.74, 1.45) | 1.32 (0.92, 1.90) |
| Top quartile | **1.54 (1.05, 2.27)** | **1.57 (1.10, 2.26)** | 1.37 (0.95, 2.00**)** | 0.72 (0.50, 1.30) | 1.34 (0.96. 1.87) |
| Per 0.5 mmol/l increase | 1.09 (0.93, 1.27) | **1.09 (1.02, 1.17)** | **1.07 (1.02, 1.13)** | 0.98 (0.92, 1.04) | 1.05 (0.98, 1.14) |

Effect of lipid metabolism on survival in the ALS cohort.

crude = adjusted for sex and age groups

adjusted = additionally adjusted for site of onset, ALS functional rating scale revised (ALSFRS-R), body mass index, and smoking (ever)

HDL = high density lipoprotein; LDL = low density lipoprotein
