# Supplementary material for: Association of blood lipids with onset and prognosis of amyotrophic lateral sclerosis: results from the ALS Swabia registry
Source: J Neurol. 2023 Feb 28;270(6):3082–90. doi: 10.1007/s00415-023-11630-4 (PMC10193299; doi:10.1007/s00415-023-11630-4)
Supplement: Supplementary file 2 — Supplementary file2 (DOCX 19 KB) [file 415_2023_11630_MOESM2_ESM.docx]

**Co-investigators of the ALS Registry Swabia Study Group:**

| Name | Location | Role | Contribution |
| --- | --- | --- | --- |
| Frank Andres, MD | Kreiskliniken Reutlingen, Department of Neurology | Site Investigator | Patient referral to registry |
| Guy Arnold, MD | Klinikum Sindelfingen-Boeblingen, Department of Neurology | Site Investigator | Patient referral to registry |
| Hartmut Baier, MD | ZFP Suedwuerttemberg, Department of Epileptology | Site Investigator | Patient referral to registry |
| James Beattie, MD | Ostalb-Klinikum Aalen, Department of Neurology | Site Investigator | Patient referral to registry |
| Friedrich Behne, MD | ZFP Suedwuerttemberg, Department of Epileptology | Site Investigator | Patient referral to registry |
| Dietmar Bengel, MD | Oberschwabenklinik Ravensburg, Department of Neurology | Site Investigator | Patient referral to registry |
| Bracknies Vera, MD | Department of Neurology, Dietenbronn | Site Investigator | Patient referral to registry |
| Burkhard Alber, MD | Klinikum Guenzburg, Department of Neurology | Site Investigator | Patient referral to registry |
| Mathias Buttmann, MD | Caritas Krankenhaus, Bad Mergentheim, Department of Neurology | Site Investigator | Patient referral to registry |
| Silke Dempewolf, MD | Department of Neurology, Ludwigsburg | Site Investigator | Patient referral to registry |
| Wolfgang Freund, MD | Praxis Biberach | Site Investigator | Patient referral to registry |
| Thomas Gasser, MD | Universitaetsklinikum Tuebingen, Department of Neurology | Site Investigator | Patient referral to registry |
| Gerhard Hamann, MD | Klinikum Guenzburg, Department of Neurology | Site Investigator | Patient referral to registry |
| Martin Hecht, MD | Bezirkskrankenhaus Kaufbeuren, Department of Neurology | Site Investigator | Patient referral to registry |
| Bernhard Heimbach, MD | University of Freiburg, Department of Neurology | Site Investigator | Patient referral to registry |
| Birgit Herting, MD | Diakonie-Klinikum Schwaebisch Hall, Department of Neurology | Site Investigator | Patient referral to registry |
| Roman Huber, MD | Klinikum Friedrichshafen, Department of Neurology | Site Investigator | Patient referral to registry |
| Paul-Juergen Huelser, MD | Fachklinik Wangen, Department of Neurology | Site Investigator | Patient referral to registry |
| Karlheinz Huber-Hartmann, MD | Kliniken Landkreis Heidenheim, Department of Neurology | Site Investigator | Patient referral to registry |
| Eric Jüttler, MD | Ostalb-Klinikum Aalen, Department of Neurology | Site Investigator | Patient referral to registry |
| Attila Kaspar, MD | Oberschwabenklinik Ravensburg, Department of Neurology | Site Investigator | Patient referral to registry |
| Rolf Kern, MD | Klinikum Kempten, Department of Neurology | Site Investigator | Patient referral to registry |
| Hubert Kimmig, MD | Kliniken Schwenningen, Department of Neurology | Site Investigator | Patient referral to registry |
| Klötzsch, Christof, MD | Department of Neurology, Hegau-Bodensee-Klinikum Singen | Site Investigator | Patient referral to registry |
| Anastasios Chatzikonstantinou, MD | Schmieder Kliniken Allensbach | Site Investigator | Patient referral to registry |
| Thomas Klopstock, MD | LMU Munich, Department of Neurology | Site Investigator | Patient referral to registry |
| Christoph Lichy, MD | Klinikum Memmingen, Department of Neurology | Site Investigator | Patient referral to registry |
| Alfred Lindner, MD | Marienhospital Stuttgart, Department of Neurology | Site Investigator | Patient referral to registry |
| Paul Lingor, MD | TU Munich, Department of Neurology | Site Investigator | Patient referral to registry |
| Dorothee Lulé, PhD | Ulm University, Department of Neurology | Site Investigator | Patient referral to registry |
| Jens Metrikat, MD | Bundeswehrkrankenhaus Ulm, Department of Neurology | Site Investigator | Patient referral to registry |
| Oliver Meudt, MD | Klinikum Memmingen, Department of Neurology | Site Investigator | Patient referral to registry |
| Andreas Meyer, MD | Weissenau, Department of Neurology | Site Investigator | Patient referral to registry |
| Andrea Naegele, MD | Christophsbad Goeppingen, Department of Neurology | Site Investigator | Patient referral to registry |
| Klaus-Dieter Neher, MD | Vinzenz von Paul Hospital, Rottweil, Department of Neurology | Site Investigator | Patient referral to registry |
| Oliver Neuhaus, MD | Kliniken Landkreis Sigmaringen, Department of Neurology | Site Investigator | Patient referral to registry |
| Clemens Neusch, MD | Praxis EMSA Singen | Site Investigator | Patient referral to registry |
| Ludwig Niehaus, MD | Department of Neurology, Winnenden | Site Investigator | Patient referral to registry |
| Jan Raape, MD | ZFP Suedwuerttemberg, Neurologie Weissenau | Site Investigator | Patient referral to registry |
| Matthias Reinhard, MD | Kliniken Esslingen, Department of Neurology | Site Investigator | Patient referral to registry |
| Johann Rothmeier, MD | ZFP Suedwuerttemberg, Neurologie Weissenau | Site Investigator | Patient referral to registry |
| Michael Sabolek, MD | Department of Neurology, Biberach | Site Investigator | Patient referral to registry |
| Martin Schabet, MD | Department of Neurology, Ludwigsburg | Site Investigator | Patient referral to registry |
| Mario Schaeff-Vogelsang, MD | Diakonie-Klinikum Schwaebisch Hall, Department of Neurology | Site Investigator | Patient referral to registry |
| Caroline Schell, MD | Kreiskliniken Reutlingen, Department of Neurologie | Site Investigator | Patient referral to registry |
| Katharina Schütz, MD | Kliniken Schwenningen, Department of Neurology | Site Investigator | Patient referral to registry |
| Barbara Schweigert, MD | Caritas Krankenhaus, Bad Mergentheim, Department of Neurology | Site Investigator | Patient referral to registry |
| Norbert Sommer, MD | Christophsbad Goeppingen, Department of Neurology | Site Investigator | Patient referral to registry |
| Mark Stroick, MD | Klinikum Memmingen, Department of Neurology | Site Investigator | Patient referral to registry |
| Mathis Synofzik, MD | Universitaetsklinikum Tuebingen, Department of Neurology | Site Investigator | Patient referral to registry |
| ThomasTrottenberg, MD | Department of Neurology, Winnenden | Site Investigator | Patient referral to registry |
| Hayrettin Tumani, MD | Department of Neurology, Dietenbronn | Site Investigator | Patient referral to registry |
| Jens Volkmann, MD | University of Wuerzburg, Department of Neurology | Site Investigator | Patient referral to registry |
| Markus Weiler, MD | University of Heidelberg, Department of Neurology | Site Investigator | Patient referral to registry |
| Wolfgang Wick, MD | University of Heidelberg, Department of Neurology | Site Investigator | Patient referral to registry |
| Christian Opherk, MD | Klinikum am Gesundbrunnen Heilbronn, Department of Neuroloy | Site Investigator | Patient referral to registry |
| Bernhard Hemmer, MD | TU Munich, Department of Neurology | Site Investigator | Patient referral to registry |
| Cornelius Weiller, MD | University of Freiburg, Department of Neurology | Site Investigator | Patient referral to registry |
| Daniel Zeller, MD | University of Würzburg, Department of Neurology | Site Investigator | Patient referral to registry |
| Jessica Baumgärtner, MD | BKH Augsburg, Department of Psychiatry | Site Investigator | Patient referral to registry |
| Christoph Born, MD | Psychiatrie Schwäbisch Hall & PMU Nuremberg, Department of Psychiatry | Site Investigator | Patient referral to registry |
| Martin Bürgy, MD | Klinikum Stuttgart, Department of Psychiatry | Site Investigator | Patient referral to registry |
| Bernhard Connemann, MD | Uniklinik Ulm, Department of Psychiatry III | Site Investigator | Patient referral to registry |
| Elmar Etzersdorfer, MD | Furtenbachkrankenhaus Stuttgart, Department of Psychiatry | Site Investigator | Patient referral to registry |
| Hubertus Friederich, MD | ZfP Zwiefalten, Departmement of Geriatric Psychiatry | Site Investigator | Patient referral to registry |
| Maximilian Gahr, MD | Uniklinik Ulm, Department of Psychiatry III | Site Investigator | Patient referral to registry |
| Alex Gogolkiewicz, MD | ZfP Zwiefalten, Departement of Psychiatry | Site Investigator | Patient referral to registry |
| Ralf Greber, MD | Vinzenz v. Paul Hospital Rottweil, Department of Geriatric Psychiatry | Site Investigator | Patient referral to registry |
| Jochen Gebhardt, MD | ZfP Wiesloch, Department of Geriatric Psychiatry | Site Investigator | Patient referral to registry |
| Heinz Grunze, MD | Psychiatrie Schwäbisch Hall & PMU Nuremberg, Department of Psychiatry | Site Investigator | Patient referral to registry |
| Karsten Henkel, MD | Christophsbad-Göppingen, Departement of Geriatric-Psychiatry | Site Investigator | Patient referral to registry |
| Walter Hewer, MD | Christophsbad Göppingen, Department of Geriatric-Psychiatry | Site Investigator | Patient referral to registry |
| Andreas Raether, MD | Klinikum Schloss Winnenden, Department of Geriatric Psychiatry | Site Investigator | Patient referral to registry |
| Andreas Joos, MD | Kliniken Schmieder Gailingen, Department of Psychotherapeutic Neurology | Site Investigator | Patient referral to registry |
| Matthias Köhler, MD | ZfP Zwiefalten, Departement of Geriatric Psychiatry | Site Investigator | Patient referral to registry |
| Ralf Kozian, MD | Vinzenz v. Paul Hospital Rottweil, Department of Geriatric Psychiatry | Site Investigator | Patient referral to registry |
| Christoph Laske, MD | Uniklinik Tübingen, Departement of Geriatric Psychiatry | Site Investigator | Patient referral to registry |
| Alexandros Michaelides, MD | Furtenbachkrankenhaus Stuttgart, Department of Psychiatry | Site Investigator | Patient referral to registry |
| Matthias Munk, MD | Uniklinik Tübingen, Departement of Geriatric Psychiatry | Site Investigator | Patient referral to registry |
| Andreas Niestroj, MD | ZfP Wiesloch, Departement of Geriatric Psychiatry | Site Investigator | Patient referral to registry |
| Matthias Ruchsow, MD | Christophsbad Göppingen, Department of Psychiatry | Site Investigator | Patient referral to registry |
| Max Schmauss, MD | BKH Augsburg, Department of Psychiatry | Site Investigator | Patient referral to registry |
| Katrin Schoeneberger-Stroick, MD | BKH Memmingen, Department of Psychiatry | Site Investigator | Patient referral to registry |
| Kai-Uwe Schoerner, MD | Kliniken Schmieder Gailingen, Department of Psychotherapeutic Neurology | Site Investigator | Patient referral to registry |
| Stefan Spannhorst, MD | Klinikum Stuttgart, Department of Mental Health and Geriatry | Site Investigator | Patient referral to registry |
| Raimund Steber, MD | BKH Memmingen, Department of Psychiatry | Site Investigator | Patient referral to registry |
| Christine Thomas, MD | Klinikum Stuttgart, Department of Mental Health and Geriatry | Site Investigator | Patient referral to registry |
| Nenad Vasic, MD | Christiophsbad Göppingen, Department of Psychiatry | Site Investigator | Patient referral to registry |
